# Efficacy, Safety, and Economic Impact of Cytisinicline Maintenance Therapy in Patients Who Are Candidates for Smoking Cessation: Protocol for a Phase IV, Multicenter, Randomized, Open-Label, Controlled, Parallel Clinical Trial (CITISILONG Trial)

**DOI:** 10.2196/76815

**Published:** 2026-01-23

**Authors:** Carlos Rabade Castedo, Ana Estany-Gestal, Carlos A Jiménez Ruiz, José Ignacio de Granda-Orive, Juan Antonio Riesco-Miranda, María Isabel Cristóbal Fernández, Angela Ramos-Pinedo, Jaime Signes-Costa Miñana, María Inmaculada Gorordo-Unzueta, Agustin Valido-Morales, Jacobo Sellarés -Torres, Eva Cabrera-César, Alejandro Frino-García, Luis Valdés Cuadrado

**Affiliations:** 1 Department of Pulmonology Complejo Hospitalario Universitario de Santiago Santiago de Compostela Spain; 2 Health Research Institute Santiago de Compostela, La Coruña Spain; 3 Research Methodology Unit Health Research Institute Santiago de Compostela, Galicia Spain; 4 Smoking Cessation Unit Hospital Clínico San Carlos Madrid Spain; 5 Department of Pulmonology Hospital Universitario 12 De Octubre Madrid Spain; 6 Department of Medicine Universidad Complutense de Madrid Madrid Spain; 7 Department of Pulmonology Hospital San Pedro de Alcántara Cáceres Spain; 8 Extremadura University Institute of Biosanitary Research Cáceres Spain; 9 Centre for Networked Research on Respiratory Diseases (CIBERES) Madrid Spain; 10 Department of Pulmonology Hospital Universitario Fundación Alcorcón Madrid Spain; 11 Department of Pulmonology Hospital Clínico Universitario de Valencia Valencia Spain; 12 INCLIVA Health Research Institute Valencia Spain; 13 Department of Pulmonology Hospital Universitario Galdakao-Usánsolo Bilbao Spain; 14 Biobizkaia Health Research Institute Bilbao Spain; 15 Department of Pulmonology Hospital Universitario Virgen Macarena Valencia Spain; 16 Department of Pulmonology Hospital Clínic de Barcelona Barcelona Spain; 17 Department of Pulmonology Hospital Clínico Universitario Virgen de la Victoria Málaga Spain; 18 Department of Medicine Universidade de Santiago de Compostela Santiago de Compostela Spain

**Keywords:** cytisinicline, smoking cessation, maintenance therapy, efficacy, safety

## Abstract

**Background:**

Cytisinicline has proven to be an effective, efficient, and safe molecule in smoking cessation. However, the established 25-day regimen could be insufficient in a high percentage of smokers, so it is necessary to study maintained therapies of this drug.

**Objective:**

This study aims to compare the efficacy of the cytisinicline regimen used in routine clinical practice versus 2 maintained regimens of 50 and 75 days, respectively. In addition, the safety and economic impact of each regime will be determined.

**Methods:**

A prospective, multicenter, open-label, controlled, parallel, phase IV clinical trial of 402 smoker patients prepared to quit smoking. The study was conducted in 10 hospitals in Spain. A control group is compared to 2 intervention groups in which the duration of the drug is increased without increasing its dose, administering half and all, respectively, of an additional marketed container that includes 100 tablets. Thus, participants will be randomized to three groups in a 1:1:1 ratio to receive cytisinicline: (1) a control group treated with cytisinicline according to the usual clinical guidelines and product information (25 days); (2) a group with a 50-day cytisinicline regimen (an additional 25 days at a dose of 1.5 mg every 12 hours), seeking to increase its efficacy while minimally impacting adherence; and (3) a group with a 75-day regimen (an additional 50 days at a dose of 1.5 mg every 12 hours), attempting to increase its efficacy, although the longer duration of the drug may threaten adherence. Efficacy in the 3 arms will be analyzed through sustained abstinence at 6 and 12 months, point abstinence rate assessed every 7 days, and abstinence rate from Day 25 to Day 50 and from Day 25 to Day 75 in the 3 study arms. (1) The variation in withdrawal and craving symptoms in the 3 groups, (2) safety through the percentage of adverse events in the 3 treatment arms, and (3) economic impact by evaluating the cost-effectiveness and cost-utility ratios of the 2 prolonged regimens versus the usual clinical cytisinicline regimen. To calculate the differences between the 3 groups for each outcome variable, a univariate analysis will be performed. Statistically significant variables will be included in a multivariate model.

**Results:**

Recruitment for the trial and patient enrollment were completed in November 2026. Follow-up of all participants will extend to December 2027.

**Conclusions:**

In conclusion, this study evaluates the optimization of cytisinicline in daily clinical practice, increasing the benefits of its pharmaceutical properties without affecting patient safety. All of this will improve the effectiveness of smoking cessation by reducing the number of smokers, which implies lower morbidity and mortality and lower costs associated with smoking.

**Trial Registration:**

European Clinical Trials Register 2024-518936-36-00; https://euclinicaltrials.eu/ctis-public/view/2024-518936-36-00

**International Registered Report Identifier (IRRID):**

PRR1-10.2196/76815

## Introduction

### Background

Cytisine (natural alkaloid), also called cytisinicline (marketed form), is extracted from a golden rain tree (*Cytisus Laburnum*) and has adverse effects similar to those reported for nicotine [1,2]. It is a partial agonist of the α4 β2 nicotinic receptors, which, when stimulated, lead to a moderate release of dopamine, contributing to the control of withdrawal symptoms. On the other hand, its antagonistic action blocks the receptor, preventing nicotine from binding to it, thus reducing the reward produced by smoking [1-4]. This duality makes cytisinicline a drug of choice for the treatment of smoking. Current scientific evidence has shown it to be a safe and effective smoking cessation molecule [5].

According to the data sheet [6] the adverse effects attributable to the use of cytisinicline, at standard doses, are mild and infrequent (<20%), with the most frequent (<20%) being gastrointestinal, dry mouth, nausea, dyspepsia, and abdominal pain. Cytisinicline is marketed as an over-the-counter drug in other European countries such as Poland, where it has been used by more than 7 million smokers, and pharmacovigilance data over the more than 50 years it has been on the market have not identified any serious or life-threatening adverse effects in smokers [7-17].

West et al [10] demonstrated that cytisinicline triples the chances of smoking cessation, and in 2 meta-analyses [11,12], where more than 4000 individuals were studied, a relative risk of 1.59 (95% CI 1.43-1.75) and 1.74 (95% CI 1.38-2.19) was found compared to placebo. These data are corroborated by new meta-analyses and systematic reviews [13-15].

In trials where it was compared with other drugs, cytisinicline demonstrated greater efficacy, measured in time to first relapse, compared with nicotine patch monotherapy [18]. Courtney et al [19], in a noninferiority trial, demonstrated that point abstinence at 4 weeks was 42.5% in the cytisinicline group vs 32.3% for varenicline, although noninferiority of cytisinicline vs varenicline could not be demonstrated at 6 months.

In Spain, cytisinicline is introduced as a smoking cessation drug in 2022 and starts to be funded by the National Health System in early 2023. The drug has demonstrated a high level of patient satisfaction and tolerability [20].

In addition, several authors have proposed various strategies to increase the efficacy/effectiveness of cytisinicline without increasing adverse effects. Rigotti et al [21], in a randomized, double-blind, placebo-controlled trial involving 810 smokers, compared 2 different durations of cytisinicline (6 or 12 weeks) versus placebo, with follow-up up to 24 weeks. For the 6-week course of cytisinicline, continuous abstinence rates were 25.3% vs 4.4% during Weeks 3-6 (odds ratio [OR] 8, 95% CI 3.9-16.3; *P*<.001) and 8.9% vs 2.6% during Weeks 3-24 (OR 3.7, 95% CI 1.5-10.2; *P*=.002). For the 12-week course of cytisinicline, continuous abstinence rates were 32.6% vs 7% during weeks 9-12 (OR 6.3, 95% CI 3.7-11.6; *P*<.001) and 21.1% vs 4.8% during weeks 9-24 (OR 5.3, 95% CI 2.8-11.1; *P*<.001). Nausea, abnormal dreams, and insomnia occurred in less than 10% of each group. Sixteen participants (2.9%) discontinued cytisinicline due to an adverse event [21,22]. The Smoking cessation with cytisinicline (SMILE) clinical trial [23] was conducted in patients in a lung cancer screening program. The study aimed to compare a 40-day cytisinicline program with an 84-day program. However, the study did not reach an adequate sample size to demonstrate a significant benefit associated with prolonged treatment. However, the data showed that the adjusted OR of 12-month continuous abstinence between standard and prolonged treatment was higher with prolonged treatment: OR 1.5 (95% CI 1-2.3).

Another clinical trial evaluated maintenance therapy with cytisinicline (cytisinicline at a standard dose for 25 days plus two 1.5 mg tablets per day for maintenance from day 26 until 12 weeks of treatment) versus varenicline at a standard dose for 12 weeks, with no difference at 6 months follow-up [24]. That is, a prolonged 3-month course of cytisinicline could be as effective as other smoking cessation drugs such as varenicline without being associated with adverse effects.

In many countries, cytisinicline is prescribed as a single 25-day course. Studies to date show that the current 25-day cytisinicline regimen is better tolerated and has fewer side effects [20]. However, there is a patient profile in whom this 25-day cytisinicline regimen is not sufficient to control cravings and withdrawal symptoms, increasing the likelihood of relapse, reducing the chances of quitting smoking, and discouraging these smokers from making another serious quit attempt with an effective drug. Recent data from Spain on experience in routine clinical practice with a 25-day cytisinicline regimen show a high rate of relapse after completing treatment. Twenty percent of smokers treated with cytisinicline at this regimen resume smoking from the end of treatment until 2 months after follow-up [25]. Establishing an optimization strategy based on prolonging treatment could increase the drug’s efficacy without being associated with a greater number of side effects, generating a favorable economic impact by increasing the number of smokers who permanently quit. We hypothesize that there is a correlation between increasing the duration of cytisinicline and its efficacy, without being associated with a greater number of adverse events/side effects.

Therefore, we aim to compare the efficacy, safety, and economic impact of this treatment regimen used in routine clinical practice in Spain versus 2 proposed extended treatment regimens (double and triple duration). Different drugs are not compared, but rather 3 different treatment regimens (25, 50, and 75 days) of the same drug (cytisinicline), one of which is the treatment regimen used in daily clinical practice.

### Objectives

The overall objective of this study is to evaluate the efficacy, safety, and economic impact of 2 extended cytisinicline regimens (50 and 75 days duration) and compare them with the standard regimen (25 days).

The primary objective is to determine the efficacy of the maintained cytisinicline regimens (50 and 75 days) versus the standard 25-day regimen.

The secondary objectives are (1) to determine the percentage and number of adverse effects in each of the 3 treatment arms and (2) to analyze the economic impact of the maintained regimens versus the standard regimen in terms of cost-effectiveness and cost-utility.

## Methods

### Trial Design

Phase IV randomized, open-label, controlled, parallel, multicenter clinical trial in patients who are candidates for smoking cessation to demonstrate the superiority of maintenance therapy with cytisinicline compared to the standard regimen.

The trial was designed according to the standardized guide for interventional trial protocols, SPIRIT (Standard Protocol Items: Recommendations for Interventional Trials statement [Multimedia Appendix 2]). A total of 402 patients from 10 smoking cessation units in Spain will be randomized. Recruitment will last for one year, with a follow-up of 52 weeks per patient recruited. Candidate patients who sign the informed consent form will be randomized to 3 possible arms in a ratio of 1:1:1.

Arm A: control group. Participants randomized to this group will be treated with cytisinicline as stated in the data sheet. They must remain smoke-free from day 5 of treatment.

Arm B: maintained therapy 1. Following the standard regimen, these patients will maintain treatment for an additional 25 days at a dose of 1.5 mg twice daily.

Arm C: maintained therapy 2. Following the standard regimen, these patients will maintain treatment for an additional 50 days at a dose of 1.5 mg twice daily (see Table 1).

**Figure 1:**
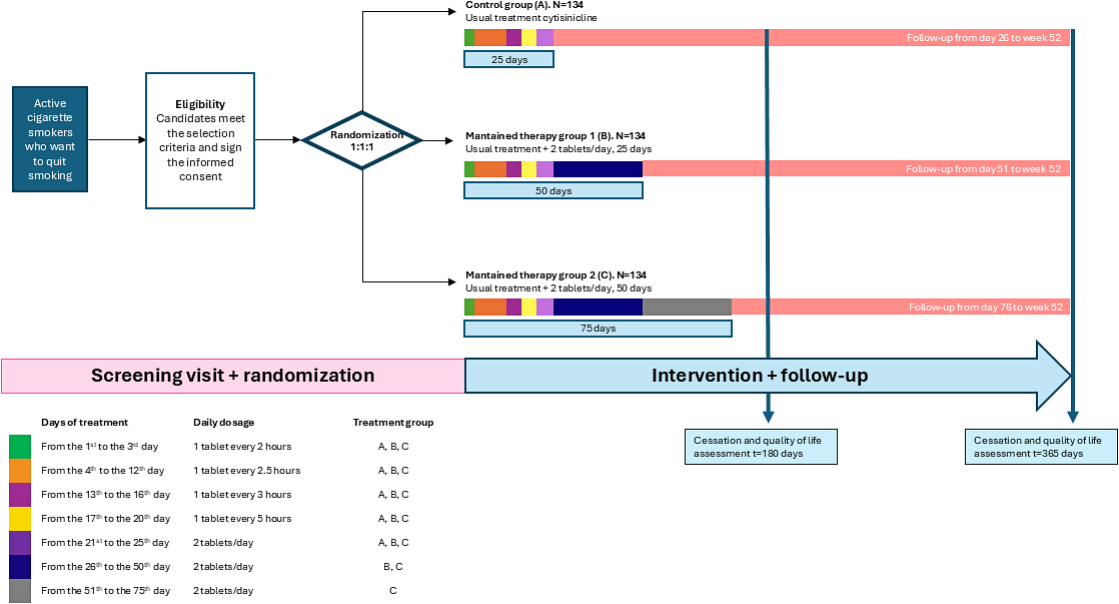
Trial design. Recruitment will last for one year, with a follow-up of 52 weeks per patient recruited.

At the end of the treatments, participants will continue in the follow-up phase, without treatment, until completing 52 weeks in the study (see Figure 1).

**Table 1:** Dosage guidelines in each of the 3 treatment arms.

Days of treatment		Recommended dosage	Maximum daily dose	Recommendations	
**Arm A: control group**					
	From the 1st to the 3rd day	1 tablet every 2 hours	6 tablets	—^a^	
	From the 4th to the 12th day	1 tablet every 2.5 hours	5 tablets	No smoking from the 5th day	
	From the 13th to the 16th day	1 tablet every 3 hours	4 tablets	No smoking	
	From the 17th to the 20th day	1 tablet every 5 hours	3 tablets	No smoking	
	From the 21st to the 25th day	2 tablets a day	Up to 2 tablets a day	No smoking	
**Arm B: maintained therapy 1**					
	From the 1st to the 3rd day	1 tablet every 2 hours	6 tablets	—	
	From the 4th to the 12th day	1 tablet every 2.5 hours	5 tablets	No smoking from the 5th day	
	From the 13th to the 16th day	1 tablet every 3 hours	4 tablets	No smoking	
	From the 17th to the 20th day	1 tablet every 5 hours	3 tablets	No smoking	
	From the 21st to the 25th day	2 tablets a day	Up to 2 tablets a day	No smoking	
	From the 26th to the 50th day	2 tablets a day	Up to 2 tablets a day	No smoking	
**Arm C: maintained therapy 2**					
	From the 1st to the 3rd day	1 tablet every 2 hours	6 tablets	—	
	From the 4th to the 12th day	1 tablet every 2.5 hours	5 tablets	No smoking from the 5th day	
	From the 13th to the 16th day	1 tablet every 3 hours	4 tablets	No smoking	
	From the 17th to the 20th day	1 tablet every 5 hours	3 tablets	No smoking	
	From the 21st to the 25th day	2 tablets a day	Up to 2 tablets a day	No smoking	
	From the 26th to the 50th day	2 tablets a day	Up to 2 tablets a day	No smoking	

^a^Not applicable.

### Study Population

This study will be conducted in the Smoking Cessation Units (SCU) of 10 Spanish hospitals with different levels of complexity of care that provide care to active smokers who wish to quit smoking. An SCU is defined as a health care service integrated within a health area that promotes, coordinates, and implements actions to prevent and treat smoking in that area, in direct collaboration with the area’s health care services. The main objectives of SCUs are clinical care, education, research, and management [26]. The SCUs are accredited by the Spanish Society of Pneumology and Thoracic Surgery and by a public agency. Their geographical distribution is representative of the Spanish population (see Table 2).

**Table 2:** Smoking Cessation Units (SCU) of 10 Spanish hospitals, with different levels of complexity of care, that provide care to active smokers who wish to quit smoking.

Recruitment centers	Estimated number of patients
University Clinical Hospital of Santiago de Compostela	42
Galdakao Hospital, Bilbao	30
San Pedro de Alcántara Hospital, Cáceres	60
San Carlos Clinical Hospital, Madrid	90
October 12 University Hospital, Madrid	40
Clinic Hospital, Barcelona	30
Alcorcón Foundation Hospital, Madrid	30
Virgen de la Macarena University Hospital, Sevilla	40
University Clinical Hospital of Valencia	20
Virgen de la Victoria Hospital, Málaga	20

The sample size of the Cytisinicline Treatment for Smoking Cessation–Long-term Study (CITISILONG) trial (N=402) is based on a minimum number of patients for each of the treatment arms. The sample size calculation is given in the Statistical Analysis section of the Methods section.

### Recruitment

A recruitment period of 3 weeks is envisaged, where the persons in charge of the SCU participating in the study will identify patients who come to the practice with the intention to quit smoking and verify and select individuals who meet the abovementioned selection criteria. Written informed consent will be obtained at the screening visit. Once the consent is signed, the patient will have the first trial visit within a maximum of 15 days. Recruitment will be noncompetitive, and each site is allocated a predetermined number of patients (see Figure 1).

### Eligibility Criteria

Male and female smokers over the age of 18 years who have smoked more than 10 cigarettes per day during the past year and during the month before the screening visit are eligible to participate in the study. Smokers must also be prepared and motivated to quit smoking (score of 7 or higher on the visual analog scale of motivation). Smokers who have received pharmacological treatment for nicotine dependence (nicotine replacement therapy, varenicline, bupropion, or cytisinicline) within the previous 3 months are excluded from participating in the clinical trial.

In addition, exclusion criteria (Textbox 1) have been designed, taking into account the considerations of the drug label. All patients meeting all inclusion criteria and no exclusion criteria are consecutively invited to participate until the calculated sample size is obtained in each center.

**Exclusion criteria for the study.**

- Renal and/or hepatic insufficiency.
- Over 65 years of age (due to limited clinical experience).
- Hypersensitivity to the active substance or to any of the excipients.
- Unstable angina.
- Recent personal history of acute myocardial infarction or recent stroke (less than 8 weeks).
- Clinically relevant arrhythmias or uncontrolled hypertension.
- History of psychiatric illness with and without treatment (panic disorder, psychosis, bipolar disorder, and depression) within the last 12 months.
- History of alcohol or other drug abuse within the last 12 months.
- Use of tobacco products other than cigarettes, e-cigarettes, or marijuana in the past month and did not agree to abstain from using these products during study participation.
- Patients being treated with antituberculosis drugs.
- Patients being treated with drugs metabolized by CYP1A1 (cytochrome *P* 450 family 1, subfamily A, polypeptide 1) and CYP1A2 (cytochrome *P* 450 family 1, subfamily A, polypeptide 2), especially those with a narrow therapeutic margin (eg, theophylline, tacrine, clozapine, and ropinirole).
- Pregnant or breastfeeding smokers.
- Women on systemic hormonal contraceptives.

### Patient Enrollment and Randomization

All participants who consent to participate and who meet the eligibility criteria will be included in the trial and randomized. They are recorded in the data collection booklet designed specifically for this purpose.

Randomization will be performed in 2 phases. First, using a simple randomization procedure, participants are assigned to 1 of the 3 arms in a ratio of 1:1:1 (This is already mentioned in Study design).

Then, as noncompetitive recruitment is proposed, the sites are randomized (1 to 8, unrestricted) so that randomization determines where to start distributing the randomized sequence in the first phase. A randomization list will be generated using SAS 9 software (SAS Institute Inc) for Windows. This process will be carried out at the coordinating center, and each participating center will be provided with its own list according to the number of patients allocated. The aim is to achieve a balanced randomization in the treatment groups in the overall sample. There are no blinding techniques, as this is an open-label study. Blind-breaking procedures are therefore not applicable.

### Intervention

Cytisinicline is administered as 1.5 mg tablets to all patients for 25, 50, or 75 days depending on arm allocation. The drug is produced and marketed by Adamed Pharma SA ul Karowa 31a 00-324 Warsaw (Poland).

According to the data sheet for cytisinicline, the D-day will be set as the date from which the smoker intends to quit smoking. This date cannot be later than the fifth day after the start of treatment with cytisinicline. This D-day should be decided by the patient with his doctor before starting treatment.

### Criteria for Discontinuation of Treatment/Intervention

A patient will be withdrawn from the study when the investigator becomes aware that a participant is experiencing a serious or intolerable adverse event that prevents continuation of the drug study.

The patient may also be excluded from the study at the investigator’s request (eg, if the investigator believes that the participant’s health could be compromised by continued treatment with the study drug). Noncompliance is not grounds for discontinuing treatment.

If a patient becomes pregnant during the study treatment period, treatment with the study drug must be discontinued. However, the participant must remain in the study to continue with safety and other study assessments.

Discontinuation may also occur at the participant’s request if they do not wish to continue drug treatment.

### Treatment Adherence Strategies

Adherence to treatment will be determined by checking blister packs at each visit and patient declaration.

### Prohibited, Permitted, and Rescue Concomitant Medication

The following are prohibited during the research period (from the signing of informed consent until the end of the follow-up period): antituberculosis drugs, systemic hormonal contraceptives, nicotine-containing products, such as nicotine replacement therapy, and drugs metabolized by the CYP1A2 (cytochrome *P* 450 family 1, subfamily A, polypeptide 2) and CYP1A1 (cytochrome *P* 450 family 1, subfamily A, polypeptide 2) enzymes, especially those with a narrow therapeutic index (eg, theophylline, tacrine, clozapine, and ropinirole). All concomitant medications not previously mentioned are permitted. Rescue medication is not required during the study.

### Posttest Care Provisions

Once the study is completed, as this is a low-intervention trial, posttrial care is not required that differs from standard clinical practice. In this case, if the participant quits smoking, he/she will be discharged from the SCU; if not, he/she will remain a patient in the SCU, and an alternative smoking cessation treatment (change of intervention format or combination of drugs) will be considered.

### Outcomes

A full set of outcomes will be analyzed to obtain clinical information on efficacy, safety, and economic impact of maintained cytisinicline therapies versus the standard cytisinicline regimen. Outcomes are classified into efficacy, safety, and economic evaluation objectives.

### Specific Outcomes Efficacy

Different outcomes will be used to measure the efficacy of cytisine in each arm of the clinical trial.

The sustained abstinence rate will be determined in each of the three treatment arms at 6 and 12 months, based on carbon monoxide co-oximetry results in ppm. The correlation between the sustained abstinence rate in each of the three arms studied at 6 and 12 months, based on co-oximetry results, and the self-reported abstinence at 6 and 12 months will be calculated.

Additionally, point abstinence will be analyzed every 7 days in each of the 3 arms studied, based on the results obtained in the CO tests.

The percentage increase in abstinence rates will be compared with the increase in the duration of cytisinicline use. The number needed to quit smoking for one patient in each treatment group will also be calculated.

Interval abstinence will also be determined in this clinical trial. The abstinence rate will be analyzed from Day 25 to Day 50 in all 3 study groups and from Day 25 to Day 75 in all 3 study arms. The abstinence rate will also be determined from Day 25 (usual arm), Day 50 (extended arm 1), and Day 75 (extended arm 2) up to 6 and 12 months.

Relapse rates will be analyzed in each treatment arm. The frequency and percentage of relapses will be determined in those who quit smoking from Day 25 (usual arm), Day 50 (extended arm 1), and Day 75 (extended arm 2) up to 6 and 12 months in all 3 arms.

Craving and withdrawal symptoms will be assessed during the clinical trial. The mean Minnesota Nicotine Withdrawal Scale (MNWS) score and the mean craving score will be calculated at the initial in-person visit in all 3 arms, and their change from this visit will be measured on days 25, 50, and 75 in all 3 study arms.

Predictors of continued abstinence at 6 and 12 months of treatment and of relapse (clinical, anthropometric, demographic, and smoking-related variables) will be determined in all study arms.

Quality of life in each group will be calculated using the EQ-5D-5L scale score at baseline, 50 days, 90 days, 6 months, and 1 year.

### Specific Outcomes (Safety)

The impact of morbidity and mortality in each treatment arm will be evaluated, as well as the percentage of side effects reported by participants, comparing their prevalence in each group throughout the study (1 year). The percentage of adverse effects at 25, 50, and 75 days will also be determined in participants, comparing their prevalence in each group.

Furthermore, the prevalence and severity of side effects for each regimen will be compared throughout the study (1 year).

Finally, the results of cardiac, hepatic, and renal variables will be compared between each group as indicators of treatment safety in each group at 25, 50, and 75 days of treatment and throughout the entire study period (1 year).

### Specific Outcomes (Economic Evaluation)

The economic analysis of 2 prolonged cytisinicline regimens vs a standard regimen will be performed by calculating their cost-effectiveness and cost-utility ratios. The effect of treatment adherence on the cost-effectiveness and cost-utility ratios of the considered therapeutic alternatives will also be evaluated.

The effect of the different predictors of sustained abstinence at 6 and 12 months (clinical, anthropometric, and demographic variables) will be evaluated in relation to cost-effectiveness and cost-utility in the three arms of the clinical trial.

The future effects (15, 30, and 50 years after smoking cessation) will be simulated in each of the treatment arms.

Finally, a budget impact model will be constructed to analyze the potential savings for the National Health System for each of the 3 therapeutic options proposed in this clinical trial.

### Schedule of Participants

Table 3 and Figure 1 show the screening visit, follow-up visits (face-to-face and telephone), tests and quizzes to be carried out at each of the visits, and the interventions.

**Table 3:** Visiting calendar. The screening visit, follow-up visits (face-to-face and telephone), tests and quizzes to be carried out at each of the visits, as well as the intervention.

Number, day, and type of visit		Visit 1 (day 1; in person)		Visit 2 (day 7; in person)		Visit 3 (day 14; telephone)		Visit 4 (day 21; in person)		Visit 5 (day 25; telephone)		Visit 6 (day 50; telephone)		Visit 7 (day 75; telephone)		Visit 8 (day 90; in person)		Visit 9 (day 120; in person)		Visit 10 (day 150; in person)		Visit 11 (week 24; in person)		Visit 12 (week 36; telephone)		Visit 13 (week 48; in person)		Visit 14 (week 52; in person)			
**Variable registration**																															
	Sociodemographic		✓																												
	Anthropometric		✓																												
	Diagnostic		✓																												
	UISPM^a^ Test		✓																												
	Motivation		✓																												
	Reward		✓																												
	Spirometry		✓																												
	Advice		✓								✓		✓		✓		✓		✓		✓		✓		✓		✓		✓		
**Efficacy evaluation**																															
	Co-oximetry		✓		✓				✓								✓		✓		✓		✓				✓		✓		
	Statement		✓		✓		✓		✓		✓		✓		✓		✓		✓		✓		✓		✓		✓		✓		
Security assessment																															
Adverse events				✓		✓				✓		✓		✓																	
Quality of life assessment																															
EQ-5D		✓								✓		✓		✓				✓		✓		✓						✓			

^a^UISPM: Test of the Specialized Smoking Unit of the Community of Madrid.

### Data Collection Variables

With regard to patient information, a database will be designed specifically for this proposal. The medical and demographic data are as follows: age (years), sex (male/female), education level (no formal education, secondary education, or higher education) and social status (low, medium, or high). We establish cut-off points for the patient’s socioeconomic level according to the OECD (Organisation for Economic Co-operation and Development) based on the approximate monthly income level (less than €1000 (€1=US $1.16–$1.17) per month = low socioeconomic level, from €1000 to €3000 per month = medium socioeconomic level, and more than €3000 per month = high socioeconomic level). Questionnaires carried out in the SCUs are on current consumption, accumulated consumption, motivation, the quitting phase, and nicotine dependence. Smoking consumption characteristics are current number of cigarettes per day (categorized as ≥20 cigarettes per day), packs-year, previous quit attempts (previous attempts, previous attempts in the last year, previous attempts with drug treatment, and previous attempts with varenicline), and graded degree of smoking (mild, moderate, severe, or very severe) [27,28].

The different characteristics and clinical profiles of each smoker will be determined by the Test of the Specialized Smoking Unit of the Community of Madrid. It consists of 7 domains (stimulation, automaticity, sedation, psychological, gestural and social dependence, and automaticity). It allows for a more detailed identification of the smoker’s specific characteristics to optimize psychological and pharmacological treatment [27].

Nicotine dependence will be determined using the Fagerström test. It provides information about the patient’s physical dependence on nicotine. It is a questionnaire of 6 questions. A score of 7 or more indicates high nicotine dependence [29].

The reward associated with tobacco use will be measured in the clinical trial. We will use the reward test for this purpose. It indicates whether the smoker smokes for pleasure (positive reward), smokes to control withdrawal symptoms (negative reward), or both (mixed reward) [30].

The analysis of withdrawal symptoms in smokers will be performed using the MNWS. It allows for the detection of the intensity of withdrawal symptoms and their variation throughout the smoking cessation follow-up process [30].

Other qualities of the smoker, such as the degree of motivation and self-efficacy, will be evaluated by the Analogue Visual Scale. This scale numerically identifies a smoker’s level of motivation and self-efficacy to quit smoking. A score above 7 indicates high motivation and self-efficacy to quit smoking [27].

The EQ-5D scale will be used in this clinical trial to assess the quality of life of smokers and its changes during the smoking cessation process. This scale measures the health status of smokers using 5 dimensions, including mobility, self-care, activities of daily living, pain and discomfort, and anxiety and depression. Participants score from 0 to 100 in each domain [31].

The urge to smoke in this study will be analyzed using the Craving Rate Score. This scale assesses anxiety or intense desire to smoke at baseline and during follow-up. It is scored from 0 to 4 [32].

The additional tests that will be performed on smokers participating in this trial include electrocardiogram, carbon monoxide levels, complete blood count, and a biochemical profile that includes liver enzymes, electrolytes, and renal function tests. Co-oximetry allows for the objective identification of smoking status, as well as confirmation of abstinence at each participant visit. A participant is identified as a nonsmoker when their CO levels measured by co-oximetry are below 10 ppm (Textbox 2).

**Efficacy and safety variables of cytisinicline.**

To analyze the efficacy of cytisinicline we used the following variables:

1. Continuous abstinence rate at six months of treatment
2. Efficacy of the maintained cytisinicline regimens (50 and 75 days) compared to the usual 25-day regimen calculated every 7 days
3. Intervention interval effectiveness
  Abstinence rate between days 25 and 50
  Abstinence rate between days 25 and 75
  Abstinence rate between days 25 and 6 months calculated every 7 days
4. Analysis of withdrawal syndrome
  MNWS score at each visit
  Variation in MNWS score between visits
  Mean MNWS score at each visit
5. Craving Analysis
  SRC score at each visit
  Change in SRC score at each visit
  Average SRC score at each visit

To analyze the safety of cytisinicline, we used the following variables:

1. Number and percentage of self-reported adverse events at the baseline visit
2. Number and percentage of self-reported adverse events on days 7, 15, 25, 50, and 75
3. Number and percentage of mild, moderate, or severe adverse events on days 7, 15, 25, 50, and 75
4. Number and percentage of serious adverse events on days 7, 15, 25, 50, and 75
5. Number and percentage of the following adverse events: nausea, vomiting, constipation, abdominal distension, abdominal pain, epigastric pain, nightmares, insomnia, parasomnias, headache, and dizziness
6. Percentage of patients with electrocardiographic abnormalities at the initial visit
7. Percentage of patients with electrocardiographic alterations at 25, 50, and 75 days
8. Percentage of patients with electrocardiographic alterations at 25, 50, and 75 days
9. Percentage of patients with abnormal liver enzyme levels at the baseline visit
10. Percentage of patients with abnormal liver enzyme levels at 25, 50, and 75 days
11. Percentage of patients with abnormal electrolyte levels at the baseline visit
12. Percentage of patients with abnormal electrolyte levels at 25, 50, and 75 days
13. Percentage of patients with abnormal renal function at the baseline visit
14. Percentage of patients with abnormal renal function at 25, 50, and 75 days

### Trial Monitoring

As this is a low-intervention trial, the monitoring and quality control processes of the generated database will be carried out by the principal investigators of each site or by a collaborating investigator of the team to whom this task is delegated.

### Pharmacovigilance

As this is a low-intervention study, the expected adverse events are those reflected in the drug label. The definition of adverse events, the classification of their severity, and the actions to be taken by the principal investigator and sponsor in the event of an adverse event are included in Multimedia Appendix 1. In all cases, the sponsor should promptly communicate to the investigators any important information that could adversely affect the safety of the participants or the conduct of the trial. The communication of such information should be concise and practical. The communication shall be in accordance with the criteria and procedure specified in the European Commission’s guidelines. The sponsor shall report to the Spanish Agency for Medicinal Products and Health Products all suspected serious and unexpected adverse reactions associated with the investigational medicinal products of which he/she has become aware. An adverse event is an undesirable experience that affects a patient during a clinical trial or study, whether or not it is considered to be related to the investigational product.

A serious adverse event is one that is life-threatening, causes persistent disability, or requires prolonged hospitalization of the patient. Apart from these, congenital anomalies and malignant neoplasms shall always be considered serious.

A nonserious adverse event is any adverse event that does not meet any of the above criteria.

An unexpected adverse event is an experience that is not described in the investigator’s manual (by nature, severity, or frequency occurred in the clinical trial).

### Statistical Analysis

#### Sample Size Determination

Based on clinical experience, it is estimated that the efficacy of cytisinicline prescribed in accordance with the data sheet is 30% (*P*^1^=.30), and that this efficacy will be greater if the maintenance regimen is extended over time. In this sense, it is hypothesized that the intermediate maintenance regimen could achieve an efficacy of 45% (*P*^2^=.40) and with the longest regimen, 50% sustained abstinence could be achieved (*P*^3^=.50). To obtain 80% power (pow=0.80) to detect differences at a 5% significance level (α=.05), 110 patients would need to be included in each arm. A loss rate of 21% is assumed, giving a final sample size of 402 patients (N=402).

#### Population of Analysis

Efficacy and safety studies shall be conducted in the entire clinically evaluable population.

#### Analysis of Demographic and Baseline Characteristics

Demographic and baseline characteristics are summarized for each study group using descriptive statistics. For continuous variables, data on the number of observations, mean, SD, minimum and maximum, or median and IQR will be presented, depending on the fit of the variable to a normal distribution. Discrete variables will be presented with frequency and percentage. Appropriate inference tests chosen according to the nature of the variables were performed to compare these characteristics between the groups under study.

### Efficacy Analysis

To determine the main objective of the work, the efficacy variable will be constructed as a dichotomous variable, resulting from an algorithm that will be constructed for this purpose once all the data from the study have been obtained. To study the causality between efficacy and treatment prolongation, a multivariate logistic regression will be constructed. To determine candidate variables, candidate variables will first be tested in a univariate regression. Those variables whose statistical significance has a *P* value of <.20 will become part of a multivariate model. The model that best explains causality will be determined using a measure of relative statistical model quality, the Akaike information criterion. The risk measure to be calculated is the relative risk with its 95% CI. Interim follow-up assessments will be analyzed using a survival analysis to determine the duration of abstinence after the smoking cessation date.

### Safety Analysis

The frequency and percentage of occurrence of adverse reactions shall be determined and categorized according to type/intensity. Calculate whether there are differences in each group compared to what is expected according to the product label.

To examine differences in the data between groups defined by the presence/absence of adverse events, the chi-square test will be applied for qualitative variables and the *t* test or the Mann-Whitney *U* test for quantitative variables. Finally, the causal relationship between safety, defined by the absence of adverse events, and treatment prolongation will be verified using multivariate logistic regression. To determine candidate variables, those with a statistical significance of *P*<.20 will be tested and included in a multivariate model.

All analyses will be performed with the statistical package SPSS (version 19.0; IBM Corp), and statistically significant values will be considered those whose *P* value is <.05.

### Economic Impact Analysis

For the economic impact analysis, direct costs (medical and nonmedical) and indirect costs will be included.

Health outcomes will be quantified in terms of quality-adjusted life years in our economic evaluation. The health-related quality of life index (utilities) for these outcomes has been estimated using the EQ-5D-3L data for smokers and ex-smokers from the 2017 National Health Survey.

Effectiveness results will be quantified in terms of years of life lost prematurely compared to the average life expectancy for men and women.

The cost-utility analysis of administering cytisinicline at the standard regimen versus 2 sustained cytisinicline therapies will be performed using the incremental cost-utility ratio. This will be calculated as the incremental cost in Euro per additional unit of quality of life gained by one alternative compared to the other. Cost differences between the 2 alternatives/differences in quality-adjusted life years between the 2 alternatives

The cost-effectiveness analysis of administering cytisinicline at the standard regimen versus 2 cytisinicline-based therapies will be performed using the incremental cost-effectiveness ratio. This will be calculated as the incremental cost in Euro per life-year gained for one alternative compared to the other.

Differences in costs and life-years lost between the 2 alternatives will be estimated. All results will be presented for different time horizons, including the short-time (5 years), medium-time (10 years), and long-time (20 years).

To assess the uncertainty of the results, univariate and multivariate deterministic sensitivity analyses will be conducted under the following assumptions: the first scenario, univariate deterministic sensitivity analysis, will consider reducing only the efficacy rate of cytisinicline by 50% while maintaining the discount rate at 3% as in the baseline analysis. The second scenario, multivariate deterministic sensitivity analysis, will consider reducing the efficacy rate by 50% but increasing the discount rate to 6%.

### Ethical Considerations

The trial will be conducted in accordance with the requirements documented in the latest version of the Declaration of Helsinki (Helsinki, 2024) and with the Standards of Good Clinical Practice. The trial is authorized for conduct by the Galician (Spain) Committee on Ethics in Medicines Research with code 2025/043 and by the Spanish Agency for Medicines and Medical Devices. It also has the agreement of the participating centers. This trial has been registered in the European Clinical Trials Register under EUDRACT code 2024-518936-36-00.

The researchers at each center will provide each participant with a patient information sheet, approved by the clinical research ethics committee, informing them about relevant study data and requesting access to their clinical and personal data recorded in their medical history and disclosed during visits. Permission is also requested for tests, examinations, and questionnaires, and the patient is informed that these actions are part of their daily clinical practice. In addition, the researcher will verbally inform the patient about the trial and answer their questions to ensure they understand the implications of their participation. The patient will be informed of their right to withdraw consent at any time and their right to have their data deleted. The patient will be informed of the possibility of being withdrawn from the study for safety reasons (an adverse event caused by the study medication) or for failure to comply with established procedures.

No patient may be included who has not given written informed consent to participate in the trial. Secondary analyses using existing data are included in the primary consent and do not require additional consent.

The research team and the sponsor of this study expressly undertake not to disclose the identity of the patients treated and to respect the confidentiality rules regarding the data and information to which they have access by participating in the trial.

In order to guarantee the confidentiality of the trial data, only the sponsor and members of the research team, the Research Ethics Committee for Medicinal Products, and the relevant health authorities will have access to them.

The research team undertakes to collect, record, and report the data accurately and correctly. The sponsor and the research team guarantee the confidentiality of the participants’ data and will ensure compliance at all times with the provisions of EU (European Union) Regulation 2016/679 of the European Parliament and of the Council of 27 April 2016 on the protection of natural persons with regard to the processing of personal data and on the free movement of such data (General Data Protection Regulation), and Organic Law 3/2018, of 5 December, on the Protection of Personal Data and Guarantee of Digital Rights.

Patients will be identified by a number in the database that guarantees complete data confidentiality, particularly the participants’ identity. Data will be sent to the study sponsor in pseudonymous form so that it is not possible to identify the patient in any way. The trial guarantees that patients cannot be identified in any of the images or supplementary material in the manuscripts obtained after this clinical trial. All information obtained as a result of this study will be considered confidential. The investigator may only report on the development and results of the study to the sponsor, the ethics committee, and the relevant health authorities.

The participant will not receive any remuneration for participating in this trial. Expenses incurred during visits related to the study will also not be covered.

## Results

Trial recruitment and patient registration will conclude in November 2026. Follow-up for all participants will continue until December 2027. At the time of this writing, we have 12 preselected patients at the sponsoring center (Smoking Unit, University Hospital Complex of Santiago de Compostela, Spain) who meet the exclusion and inclusion criteria of the study and who will begin the initial visit in the coming days.

## Discussion

### Principal Findings

Extended treatment regimens with cytisinicline of 50 and 75 days may be more effective than the 25-day treatment regimen currently used in clinical practice. Furthermore, the percentage of adverse effects and the economic cost should not be higher with extended treatment regimens. These expected results will confirm the current scientific evidence on this drug. For example, a multicenter observational study conducted in Spain with more than 300 patients treated with cytisinicline for 25 days shows a relapse rate of 20% in the 2 months following the end of treatment. For every point increase in craving at the end of treatment, the success rate of smoking cessation decreases by 13% at 6 months of follow-up [25]. In other words, the intensity of craving at 4 weeks is inversely correlated with the drug’s efficacy. Increasing the number of days of drug administration could correct this intense desire to smoke.

Two placebo-controlled clinical trials conducted in the United States compared a cytisinicline regimen at a dose of 3 mg every 8 hours for 6 weeks versus 12 weeks. These trials showed an increase in the number of smokers who quit smoking when the treatment regimen was extended beyond 6 weeks. Furthermore, the increased number of treatment days allowed a group of smokers who were gradually reducing their consumption to quit smoking, thus increasing the percentage of smokers who quit. In other words, there will be a group of smokers who will require a longer time to quit smoking [21,22]. No greater number of adverse events were observed with these prolonged regimens. In Europe, a clinical trial in a lung cancer screening program demonstrated that a prolonged cytisinicline regimen with doses different from those specified in the product information—an 84-day regimen—was more effective than the 40-day regimen, without a greater number of side effects [23].

As strengths, it is the first study to compare 3 different cytisinicline regimens: a standard 25-day regimen used in routine clinical practice approved in the product information versus 2 prolonged treatments of double and triple duration without modifying the dose or frequency of the same, unlike other studies that evaluate a higher dose, a different frequency of administration, or compare cytisinicline with other drugs [21-23].

This is a low-intervention study due to several characteristics: (1) studies of extended regimens exist in the European Union [23]. These analyze the efficacy and safety of extended cytisinicline treatments of 40 and 84 days. We also have experience with extended doses of 1.5 mg every 12 hours for 3 months [24]; (2) modifications to drug administration are minimal. One of the study arms corresponds to the regimen used in daily clinical practice; and (3) patient selection was performed according to the drug’s prescribing information. All of this ensures its feasibility.

Unlike other studies that propose a substantial change in the dose and frequency of the molecule, this one uses extended treatment regimens of an additional 25 and 50 days, with minimal modification to the commercially available drug formulation. Thus, in Spain, each pack of cytisinicline contains 100 tablets, so one-third of patients will be randomized to take half the tablets contained in the additional pack (2 tablets a day for an additional 25 days) and another third to take all of them in that pack (2 tablets a day for an additional 50 days). Furthermore, these regimens, with their lower administration frequency, could be more adherent than regimens of 3 times a day for 12 weeks.

At the time of this writing in Europe, the same dose and duration of cytisine are used for all smokers. However, smokers represent a heterogeneous patient population with differences in dependence, emotionality, motivation, and comorbidities. This study, using a multivariate model, allows the identification of the group of patients who would require extended treatment with cytisinicline, personalizing its use in routine practice.

It is a multicenter study conducted in different geographical areas of Spain, with a representative sample. Moreover, it is carried out by a research group with experience in the diagnosis and treatment of smoking cessation. Patients treated in 10 specialized smoking cessation units accredited by scientific societies are included. Furthermore, this is an independent trial, free from the financial interests of the pharmaceutical industry, which enhances the rigor of the results. Funding for disseminating the results will be obtained through grants or public funds.

This trial incorporates the calculation of the economic impact of extended doses, determining the savings for the National Health System that these regimens could generate by increasing the number of ex-smokers.

The results of the study will be published in indexed scientific journals and presented at national and international conferences.

### Limitations

This study is not placebo-controlled; therefore, the effect of the intervention (extended cytisinicline treatment) cannot be accurately determined. The results of the intervention could be attributed not only to the drug but also to external factors. However, the use of a placebo could compromise the low level of intervention in this trial, according to regulatory authorities in Spain and the European Union. Furthermore, the synthesis of the placebo would hinder the feasibility of this study. Also, not including a placebo in the 25-day and 50-day treatment arms could provide results more aligned with routine clinical practice.

On the other hand, the efficacy and safety of different durations of cytisinicline treatment are not compared to other drugs for the treatment of nicotine dependence, such as varenicline or nicotine replacement therapy. This is because this is not the ultimate goal of this study. However, such a comparative analysis will be carried out in subsequent studies. The selected patient population consists of smokers attending SCUs with high nicotine dependence and associated comorbidities and does not represent the general smoking population. This selection bias may influence the greater efficacy of extended cytisinicline treatment. However, these are units of varying complexity, and smoking cessation can also be addressed in individuals with less nicotine dependence.

Because this is a low-intervention clinical trial, smokers older than 65 years and those with serious psychiatric or cardiovascular comorbidities are not included. Therefore, we cannot generate scientific evidence on extended treatment in these vulnerable populations.

Abstinence outcomes are objectively determined during in-person visits using co-oximetry. However, other more precise methods, such as cotinine determination in body fluids, are not used. Nevertheless, this test is accepted as a powerful marker for verifying abstinence, establishing a carbon monoxide (CO in ppm) cutoff point of less than 10 ppm [27]. Although different monitors may vary in their sensitivity to CO, combining this measurement with patient self-reporting improves the predictive capacity of co-oximetry.

Furthermore, nonpharmacological interventions may differ between centers, which could affect the results. However, a standardized model of psychological counseling is established across the centers, based on behavior modification, avoidance, substitution, and cognitive techniques.

Adherence was determined by patient self-report and the number of tablets in the blister pack, not by measuring the drug in bodily fluids. However, the high frequency of follow-up visits in the study allows for better identification of adherence to cytisinicline.

### Conclusions

Cytisinicline, when used in a standard regimen, triples the likelihood of smoking cessation, as it is a well-tolerated drug with high adherence. However, the percentage of smokers who quit is less than 50%. Therefore, it is essential to design new administration methods and therapeutic regimens.

An optimization strategy for this drug, such as increasing its duration, enhances its benefits without compromising patient safety, representing a new opportunity for smokers to quit.

Furthermore, this study identifies the population of smokers susceptible to a prolonged treatment regimen with cytisinicline. All of this will lead to a reduction in the number of smokers, decreasing, in the short to medium term, morbidity, mortality, and the direct and indirect costs associated with smoking.

## Appendix

Multimedia Appendix 1 Definition and classification of adverse events. Actions to be taken by the principal investigator and the sponsor in the event of an adverse event.

Multimedia Appendix 2 SPIRIT 2025 checklist of items to address in protocol for a phase IV, multicentre, randomised, open-label, controlled, parallel, multicentre clinical trial that evaluates efficacy, safety and economic impact of citisinicline maintenance therapy in patients who are candidates for smoking cessation CITISILONG trial.

## Abbreviations

CITISILONG: Cytisine Treatment for Smoking Cessation–Long-term study
CYP1A1: cytochrome *P* 450 family 1, subfamily A, polypeptide 1
CYP1A2: cytochrome *P* 450 family 1, subfamily A, polypeptide 2
EU: European Union
MNWS: Minnesota Nicotine Withdrawal Scale
OECD: Organisation for Economic Co-operation and Development
OR: odds ratio
SCU: Smoking Cessation Unit
SMILE: smoking cessation with cytisinicline
SPIRIT: Standard Protocol Items: Recommendations for Interventional Trials Statement

## Data Availability

The datasets generated or analyzed during this study are not publicly available due to their nature as protected health information. Access to this data is restricted by the participating institutions.
