# Supplementary material for: Efficacy, Safety, and Economic Impact of Cytisinicline Maintenance Therapy in Patients Who Are Candidates for Smoking Cessation: Protocol for a Phase IV, Multicenter, Randomized, Open-Label, Controlled, Parallel Clinical Trial (CITISILONG Trial)
Source: JMIR Res Protoc. 2026 Jan 23;15:e76815. doi: 10.2196/76815 (PMC12881894; doi:10.2196/76815)
Supplement: Multimedia Appendix 1 [file resprot_v15i1e76815_app1.docx]

**PHARMACOVIGILANCE**

**Definition and classification of adverse events.**

| **How is an adverse event defined?**  An adverse event (AE) is a undesirable experience that affects a patient during a clinical trial or study, whether or not it is considered to be related to the investigational product.  **Classification of adverse events**  A serious adverse event (SAE) is one that is life-threatening or life-threatening, causes persistent disability, or requires prolonged hospitalisation of the patient. Apart from these, congenital anomalies and malignant neoplasms shall always be considered serious.  A non-serious adverse event is any adverse event that does not meet any of the above criteria.  An unexpected adverse event is an experience that is not described in the investigator's manual (by nature, severity, or frequency |
| --- |

**Actions to be taken by the principal investigator and the sponsor in the event of an adverse event.**

| All adverse events, whether observed by the investigator, obtained by questioning the patient, or voluntarily reported by the patient, should be recorded in the eCRD with the following data: duration, pattern, severity, relationship to the test product, treatment, actions taken in relation to the test product, and outcome.  The investigator reporting the event shall determine the causal relationship of an adverse event to the IMP.  In the absence of information on possible causality from the investigator reporting the event, the sponsor shall make every effort to obtain the investigator's causality assessment if it is not mentioned in the original source document.  The sponsor should not downgrade the investigator's causality assessment. In the event that the sponsor disagrees with the investigator's causality assessment, the adverse event report should reflect the opinion of both sources.  The investigator reporting the adverse event shall determine the clinical intensity by classifying the adverse event as mild, moderate or severe depending on the degree of interference with the patient's daily activities. In the absence of information on the investigator's assessment of the clinical severity of the adverse event reported, the sponsor should make every effort to obtain the investigator's assessment of the severity if it is not mentioned in the original source document.  According to the label, clinical studies and previous experience with the use of cytisynicline-containing products indicate that cytisynicline has a good tolerability. The label itself indicates that the proportion of patients who discontinued treatment due to adverse reactions was 6-15.5 %, and in controlled studies this value was similar to the proportion of patients who discontinued treatment in the placebo group. Mild to moderate adverse reactions were generally observed, most commonly affecting the gastrointestinal tract. Most adverse reactions occurred at the beginning of treatment and disappeared during treatment. It is further indicated that these symptoms could be a consequence of smoking cessation rather than the use of the medicinal product. The sponsor shall communicate without undue delay within 15 calendar days to the Spanish Agency for Medicines and Health Products and to the CEIm. Such communication shall be made in accordance with the guidelines of the European Commission or, where applicable, with the procedures laid down in the instructions for the conduct of clinical trials in Spain published by the Spanish Agency for Medicinal Products and Health Products.  The sponsor shall report to the Spanish Agency for Medicinal Products and Health Products all suspected serious unexpected adverse reactions associated with the investigational medicinal products of which he/she has become aware that have occurred in the clinical trial. In addition, suspected serious unexpected serious adverse reactions occurring outside the trial shall be reported in accordance with the criteria set out in the European Commission guidelines. In all cases, such reporting shall be done through the European database Eudravigilance. The timing of reporting of suspected serious unexpected adverse reactions by the sponsor to the Spanish Agency for Medicinal Products and Health Products shall depend on the severity of the reaction and shall be determined as follows:  (a) In the case of suspected serious unexpected fatal or life-threatening adverse reactions, as soon as possible and in any case within seven days of the sponsor becoming aware of the reaction.  (b) In the case of suspected serious unexpected non-fatal or non-life-threatening adverse reactions, no later than 15 days after the sponsor has become aware of the reaction.  (c) in the case of suspected serious unexpected fatal or life-threatening adverse reactions which were not initially considered as such, as soon as possible and in any case no later than seven days after the sponsor has become aware that the reaction is fatal or life-threatening.  Where necessary to ensure prompt notification, the sponsor may make an initial incomplete notification which shall be completed as far as possible within eight days. |
| --- |
